# The benefits of intraoperative virtual reality distraction in anterior cruciate ligament reconstruction (ACLR) under spinal anaesthesia

**DOI:** 10.1002/jeo2.70301

**Published:** 2025-06-05

**Authors:** Ahmed Mabrouk, Henri Peuchot, Christophe Jacquet, Shintaro Onishi, Matthieu Ollivier

**Affiliations:** ^1^ Department of Trauma and Orthopaedics Basingstoke and North Hampshire Hospital Basingstoke UK; ^2^ APHM, CNRS, ISM, Sainte‐Marguerite Hospital, Institute for Locomotion Aix‐Marseille University Marseille France

**Keywords:** anterior cruciate ligament, anxiety, augmented reality, virtual reality

## Abstract

**Purpose:**

Patient anxiety in anterior cruciate ligament reconstruction (ACLR) is a preventable potential risk factor for poor patient satisfaction and surgical outcomes. This study aimed to assess the impact of virtual reality (VR) on the anxiety of patients undergoing ACLR under spinal anaesthesia (SA). The primary outcome was the perioperative patient anxiety level as assessed by the State‐Trait Anxiety Inventory (STAI‐Y1) score.

**Methods:**

A prospective randomised controlled study, of a single‐centre series of patients undergoing ACLR + Lemaire lateral extra‐articular tenodesis (LET) under SA, was conducted. Patients were randomised into two groups: Group 1 was a control group, where standard ACLR was performed, and Group 2 had ACLR performed with VR headset used. Pre‐ and postoperative VAS pain, comfort and anxiety were recorded. Additionally, pre‐and postoperative patient anxiety was recorded by the State‐Trait Anxiety Inventory (STAI) Score. STAI‐Y1 score form was used, which assesses the state (current) level of anxiety. Intraoperative adverse events were recorded including sedation requirements and rescue analgesia and patient postoperative satisfaction was recorded.

**Results:**

A total of 60 patients underwent ACLR + LET under SA. Patients were randomised into 2 groups: Group 1 (*n* = 30), the control group, had standard ACLR performed. Group 2 (*n* = 30) had ACLR with the use of the VR headset. There was no difference in the mean age and mean BMI between the two groups (*p* > 0.05). Intraoperatively, less rescue analgesia and less required sedation were encountered in the VR group compared to the control group, 13.3% versus 36.7% (*p* = 0.03), and 10% versus 56.7% (*p* < 0.0001), respectively. There was no significant difference in preoperative anxiety between both groups either on the VAS or STAI‐Y1 scores (both *p* = 0.8). The preoperative STAI‐Y1 score was 33.3 ± 7.9 in the control group versus 34.9 ± 9.7 in the VR group (*p* = 0.8). Intraoperatively, there was significantly less anxiety STAI‐Y1 score in the VR group of 29.6 ± 7.3 compared to the control group at a score of 36.9 ± 5.8 (*p* < 0.0001). This continued to be the case in the immediate postoperative period, with significantly lower anxiety score STAI‐Y1 in the VR group versus the control group, 30.6 ± 6.9 versus 37.3 ± 5.7, respectively (*p* < 0.0001). High VAS satisfaction assessed by both the anaesthesiologist and anaesthetic nurse was reported in the VR group versus the control group, 80% versus 36.7% and 93.3% versus 46.7% (all *p* < 0.0001). VAS pain score was significantly less in the VR group compared to the control group; 2.3 ± 2.1 versus 3.1 ± 2.9 (*p* = 0.03) in the recovery room, and 2.9 ± 2.7 versus 3.4 ± 3 (*p* = 0.04) before discharge to home.

**Conclusion:**

Virtual reality‐augmented anterior cruciate ligament reconstruction results in significantly fewer intraoperative adverse events, significantly less intraoperative and less immediate postoperative patient's anxiety, and higher overall patient satisfaction when assessed by the anaesthetic team.

**Level of Evidence:**

Level I, randomised controlled trial.

**Abbreviations**
ACLR: anterior cruciate ligament reconstruction
ASA: American Society of Anaesthesiology
BMI: body mass index
EVAN‐LR: evaluation of anaesthesia local regional
LET: lateral extra‐articular tenodesis
SA: spinal anaesthesia
SKV: simple knee value
STAI: state‐trait anxiety inventory
VAS: visual analogue scale
VR: virtual reality

## INTRODUCTION

Intense perioperative patient anxiety impacts the requirements and effectiveness of analgesia and anaesthesia and potentially affects the incidence of complications, postoperative recovery, and overall outcomes [8, 18, 42]. It is reported that up to 80% of patients undergoing elective surgery experience anxiety [36, 44]. This is of special relevance in anterior cruciate ligament reconstruction (ACLR), where a patient's anxiety can further impact postoperative rehabilitation and return to sport [7, 9, 16, 20, 34, 45].

For over two decades, ACLR has been performed as a day‐case surgery [1, 19, 27]. Nevertheless, among the chief contraindications of same‐day discharge is postoperative pain [11, 37]. Anxiety can increase a patient's perception of the pain and its severity [31]. Hence, anxiety could also lead to a delayed discharge in cases of ACLR.

A variety of anaesthetic modalities can be employed for ACLR, either general anaesthesia, spinal anaesthesia, or quadruple nerve blockade with a myriad of combined analgesia [5]. The combined analgesia aims at reducing sedative medications and opioids which could delay same‐day discharge [5]. In the last decade, virtual reality (VR) has been introduced as an anaesthetic adjunct, for patient distraction by immersion, with the objective of reducing sedative medications [32]. A possible additional benefit of VR could be improving patients' overall surgical experience and satisfaction.

VR has demonstrated significant capability in patients' distraction from pain and reduction of perioperative anxiety, through immersion in a virtual pacifying environment, with subsequent reduction in the perceived pain and anxiety intensity [22, 29]. This could be explained by the limited attentional capacity theory, where the brain prioritises which stimuli to attend to due to its limited attentional resources, often focusing on more relevant information and filtering less relevant ones [14].

Few studies have investigated the use of VR combined with spinal anaesthesia in lower limb arthroplasty and demonstrated no significant reduction in the use of sedative medications [10, 24]. On another array, a study reported significant improvement in patient satisfaction when VR was used during hand surgery [2]. However, there are no reports in the literature on whether VR would benefit patients undergoing ACLR in terms of anxiety reduction, reduction of sedative medications, or an improvement in overall patient satisfaction.

This study aimed to assess patient anxiety intraoperatively and post‐operatively on the same day of ACLR surgery under spinal anaesthesia (SA) with or without VR distraction. The primary outcome was the postoperative patient anxiety level as assessed by State‐Trait Anxiety Inventory (STAI Y‐1) score. The secondary outcomes were intraoperative sedation requirements, complications (hypotension and oxygen need), postoperative pain and comfort scores on a visual analogue scale (VAS), and patient satisfaction. It was hypothesised that there would be no difference in the primary or secondary outcomes with or without using the VR distraction.

## METHODS

After institutional review board approval, a single‐centre prospective randomised controlled trial was conducted (APHM registration number DGR‐2020‐1261). Between the 1 January 2022 and the 1 January 2023, a total of 117 primary ACLRs were scheduled to have day‐case primary ACLR using pediculated tripled semitendinosus and Lemaire lateral extra‐articular tenodesis (LET) under spinal anaesthesia. Hundred and eight patients accepted to be involved in the study, of which the first 60 consecutive patients were enroled between the 1 January 2022 and to 28 April 2022. The inclusion criteria were patients ≥ 18 years of age who will undergo primary ACLR following a traumatic ACL injury with or without associated meniscal injury. Excluded cases were patients with concomitant knee ligamentous injury requiring surgical intervention, or concomitant cartilage or slope changing procedures. Patients who had previous knee surgeries and those with generalised anxiety disorders, were also excluded.

### Randomisation procedure

During enrolment, the VR principle and protocol were explained to all patients. Then, patients were randomised into 2 groups (each n = 30), regardless of the specifics of meniscal injury. Group 1 was a control group, where the standard institutional protocol of ACLR was employed, and group 2 had ACLR with VR headset used. A previously validated virtual reality headset (HypnoVR®) was utilised [13]. All patients' demographics were recorded, as well as the regular use of anxiolytics, alcohol, and tobacco Table 1. Before the surgery day, a group meeting involving the patient, the surgeon, and the anaesthetist was undertaken, where the principles of VR headset and intraoperative distraction were discussed, and informed patient consent was taken. All patients were informed about the study and the possibility of wearing a headset during the operation. All patients were asked to bring their earphones to listen to music or radio in case they were not selected to wear the headset.

**Table 1:** Patient demographics, anxiolytic, alcohol and tobacco use.

Variable	Control group	VR group
Age	30.6 ± 12.9	28.8 ± 9.5
	[25.7, 35.6]	[25.3, 32.4]
Gender		
Male	21 (70%)	19 (63.3%)
Female	9 (30%)	11 (36.3%)
BMI (kg/m^2^)	19.3 ± 1.4	19.1 ± 0.8
	[18.7, 19.8]	[18.8, 19.4]
ASA I	30	30
Usual anxiolytics use		
NO	24	27
YES	6	3
Usual alcohol use		
NO	23	27
YES	7	3
Usual tobacco use		
YES	25	26
NO	5	4

Abbreviations: ASA, American Society of Anaesthesiology; BMI, body mass index; VR, virtual reality.

**Figure 1:**
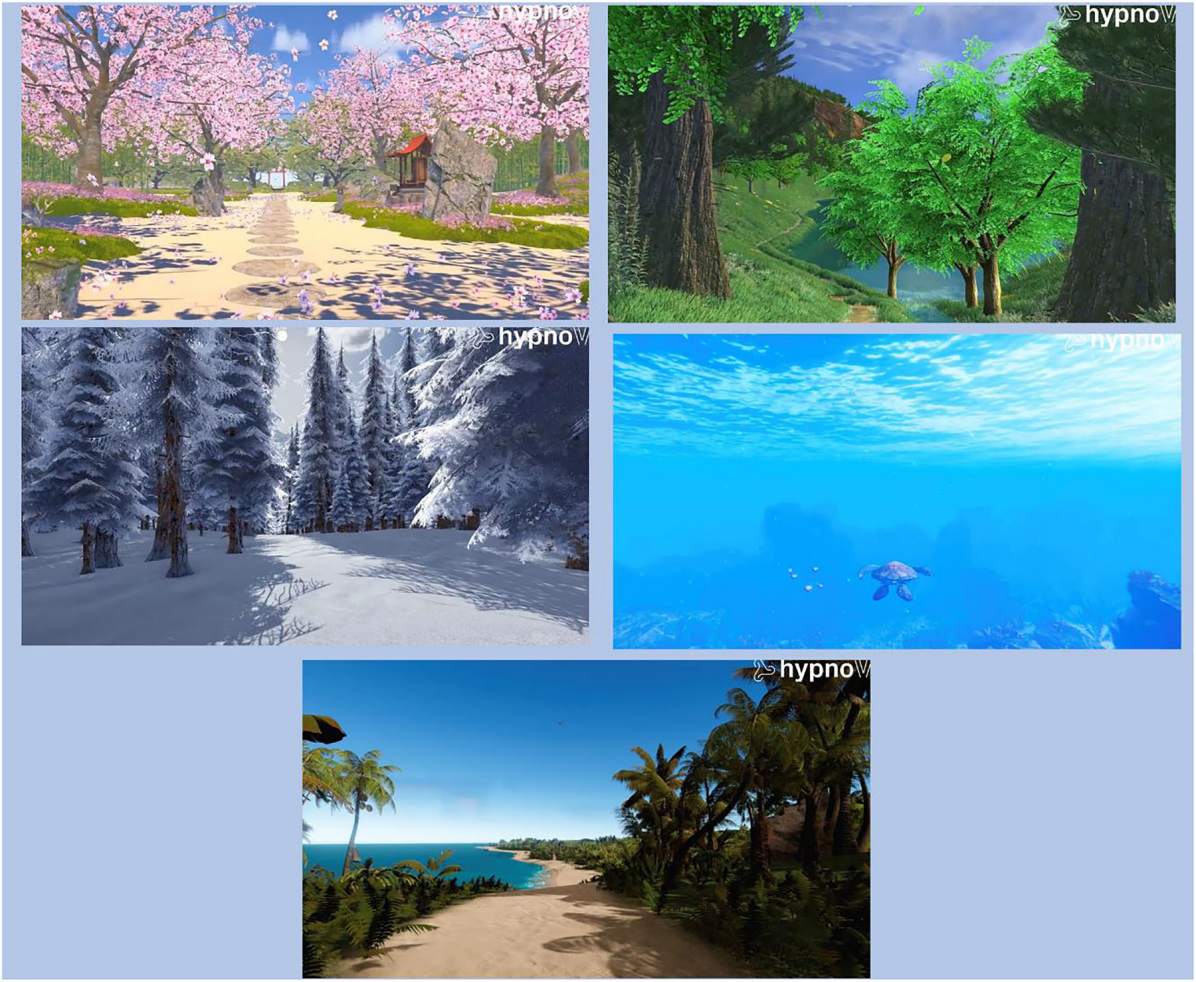
Demonstration of the screen view for the five virtual reality (VR) universes proposed for the patient on the touchpad before the surgery.

On the surgery day, patients in the control group were allowed to use their own earphones should they prefer. Whereas, patients in the VR group, had help from the anaesthesiologist to choose their preferred visual and auditory universe on a touchpad, which will be running during the surgery. A proposal of five universes was made: undergrowth, astral travel, snowy landscape, diving, and tropical beach Figure 1. Additionally, visual support with either a male or a female voice was available to play.

The process involved first attaching the headset, and then the headphones. The distraction procedure commenced during the skin preparation. The VR was used throughout the whole surgical procedure and removed after the wound dressings.

### Preoperative anaesthetic strategy

Premedication was not required for all patients. Standard monitoring was applied, including heart rate, blood pressure checks, and oxygen saturation monitoring every 10 min. Spinal anaesthesia was performed using bupivacaine and sufentanil (L3–L4 or L4–L5). Spinal anaesthesia was tested after 30 min, and once confirmed effective, a standard analgesic protocol was employed for all patients in both groups: dexamethasone 8 mg, paracetamol 1 g/6 h, profenid 100 mg/8 h IV. Droleptan was administered based on the Apfel score [3].

Additionally, all patients had an iPACK block (infiltration between the popliteal Artery and Capsule of Knee).

### Surgical strategy

In group 1, a standard institutional protocol for ACLR was undertaken. In Group 2, the headset was set up after confirming the effect of the spinal anaesthesia. All patients were examined under anaesthesia with Lachman's test and Pivot shift test and findings were recorded in Table 2. All patients were made aware that they could communicate with the medical team to suspend the distraction for any reason throughout the whole surgical procedure. Otherwise, the headset was removed after the surgery. No routine postoperative immobilisation was undertaken, unless in some cases with associated meniscal repairs.

**Table 2:** Examination under anaesthesia, associated meniscal lesions and postoperative immobilisation. Intraoperative and postoperative adverse events.

Examination under anaesthesia	Total	Control group	VR group	*p* values
Lachman's test				0.1
Garde I	8	6	7	
Grade II	37	19	18	
Grade III	8	4	4	
Missing data	7	1	1	
Pivot shift test				0.07
Grade 0	1	1	0	
Grade I	10	6	9	
Grade II	34	18	16	
Grade III	8	4	4	
Missing data	7	1	1	
Meniscal lesions				0.4
No meniscal lesions	43	20	23	
One meniscus lesion	6	3	3	
Two meniscal lesions	9	5	4	
Three meniscal lesions	2	2	0	
**Intraoperative adverse events**				
Intraoperative sedation				<0.0001
Not required	40	13	27	
Required	20	17	3	
Intraoperative rescue analgesia				0.03
YES	15	11	4	
NO	45	19	26	
Postoperative Nausea				0.4
YES	12	8	6	
NO	48	22	24	

Abbreviation: VR, virtual reality.

### Intraoperative anaesthetic strategy

Sedative medications were used if required for any patient in both groups. Propofol or remifentanil in TIVAC was used. Whenever sedation was not sufficient, a conversion to general anaesthesia was performed.

### Postoperative anaesthetic strategy

After surgery completion, all patients were recovered in the recovery room. A standard analgesia protocol was prescribed: paracetamol 1 g/6 h, profenid 100 mg/12 h (depending on renal function). In scenarios where the pain was uncontrolled (EVA > 3), rescue analgesia was used. A morphine titration was performed at 0.1 mg/kg (max 4 mg), then repeated injections every 5 min of 0.0025 mg/kg until sufficient analgesia was achieved. When the patient experienced nausea or vomiting, ondansetron 4 mg was administered.

### Details of the collected data

An evaluation of the patient's anxiety was performed using the STAI‐Y1 score proposed by Spielberger in the 1980s, whose French adaptation was made in 1993 with his collaboration [40, 41]. STAI‐Y1 has been used to assess patient's anxiety following other knee procedures [17, 35]. STAI assesses self‐reported anxiety based on current symptoms, symptoms' severity, and the overall propensity to be anxious. STAI includes two subscales: The State Anxiety Scale (S‐Anxiety) which evaluates the current subjective anxiety state and uses the form STAI‐Y1. Whereas, the Trait Anxiety Scale (T‐Anxiety) measures the liability to anxiety in a more stable state such as general states of calmness, confidence, and security, and uses the form STAI‐Y2 [26]. The minimum possible score for STAI‐Y1 is 20, whereas the maximum possible score is 80 [40, 41]. The patient's anxiety was assessed in several settings: preoperatively, intraoperatively (during skin closure), immediately postoperatively (In the recovery room), and before discharge, using the form STAI‐Y1.

Intraoperative complications such as hypotension (drop of systolic blood pressure below 70 mmHg), hypoxia (decreased oxygen saturation below 94%), or apnoea were recorded. Patient comfort, pain, and satisfaction were evaluated postoperatively with a visual analogue scale (VAS) [30]. VAS pain was recorded in the immediate postoperative period (In the recovery room) and before discharge, and VAS comfort and satisfaction were recorded only before discharge. At one year postoperatively, patients were clinically assessed with the simple knee value (SKV) score.

### Statistical analysis

Data was analysed with the SPSS 20.4 software. Descriptive data was presented as mean and standard deviations. The chi‐square test was used for categorical variables and the student's *t‐*test for continuous variables. The alpha risk was set at *p* = 0.05. A power sample calculation estimated that 20 patients per group will be necessary to distinguish differences in STAI score of 3 ± 2 with a statistical power of 80%.

## RESULTS

A total of 60 patients underwent ACLR under SA. Group 1 (*n* = 30) had standard ACLR performed. Group 2 (*n* = 30) had ACLR with the use of the VR headset. The mean age in Group 1 was 30.6 ± 12.9 years, and in Group 2 was 28.8 ± 9.5 years, and the mean BMI was 19.3 ± 1.4 kg/m^2^ in Group 1 and 19.1 ± 0.8 kg/m^2^ in Group 2, with no significant difference between both groups (*p* = 0.3 and *p* = 0.7, respectively). Also, there were no significant differences between both groups in usual anxiolytic, alcohol, and tobacco use (all *p* > 0.05). Table 1. Results of examination under anaesthesia and associated meniscal lesions are reported in Table 2 with no significant difference between both groups (all *p* > 0.05). Fifteen cases had concomitant meniscal repairs and required postoperative immobilisation in a hinged knee brace.

There was no significant difference in preoperative anxiety between both groups either on the VAS or STAI‐Y1 scores (Both *p* = 0.8), as well as no significant difference in the preoperative VAS comfort scores between both groups (*p* = 0.09) Table 3.

**Table 3:** Preoperative and postoperative VAS scores (pain, anxiety and comfort) and STAI Y‐1 scores.

VAS scores	Control group	VR group	*p* values
Preoperative scores			
VAS pain	1.1 ± 2.9	1.3 ± 2.2	0.3
Anxiety	2.8 ± 0.8 [2.5, 3.1]	3 ± 1.1 [2.6, 3.4]	0.8
Comfort	8.6 ± 1 [8.2, 9]	8.2 ± 0.8 [7.9,8.5]	0.09
Postoperative scores			
VAS pain (recovery room)	3.1 ± 2.9	2.3 ± 2.1	0.03
VAS pain (before discharge)	3.4 ± 3	2.9 ± 2.7	0.04
Anxiety	2.1 ± 1.7 [1.4, 2.7]	2.7 ± 1.4 [2.2, 3.3]	0.9
Comfort	7.5 ± 1.5 [6.9, 8]	10.1 ± 12.7 [5.4, 14.8]	0.9
STAI Y‐1 scores			
STAI Y‐1 preoperative	33.3 ± 7.9	34.9 ± 9.7	0.8
	[30.3, 36.3]	[31.2, 38.5]	
STAI Y‐1 intraoperative	36.9 ± 5.8	29.6 ± 7.3	<0.0001
	[34.8, 39.1]	[26.9, 32,3]	
STAI Y‐1 postoperative	37.3 ± 5.7	30.6 ± 6.9	<0.0001
	[35.2, 39.5]	[28, 32.2]	

Abbreviations: STAI Y‐1, state‐trait anxiety inventory; VAS, visual analogue scale; VR, virtual reality.

Adverse events were less encountered in the VR group. Intraoperatively, there was significantly less required sedation for patients in the VR group 10% versus 56.7% in the control group (*p* < 0.0001). Postoperatively, rescue analgesia was less required in 13.3% of the patients in the VR group versus 36.7% of the patients in the control group (*p* = 0.03). No apnoea episodes were reported intraoperatively or postoperatively. No intraoperative nausea was reported, and there was no inter‐group difference in the reported postoperative nausea. No intraoperative or postoperative reported hypotension or hypoxaemia Table 2. VAS pain score was significantly less in the VR group compared to the control group; 2.3 ± 2.1 versus 3.1 ± 2.9 (*p* = 0.03) in the recovery room, and 2.9 ± 2.7 versus 3.4 ± 3 (*p* = 0.04) before discharge to home.

**Figure 2:**
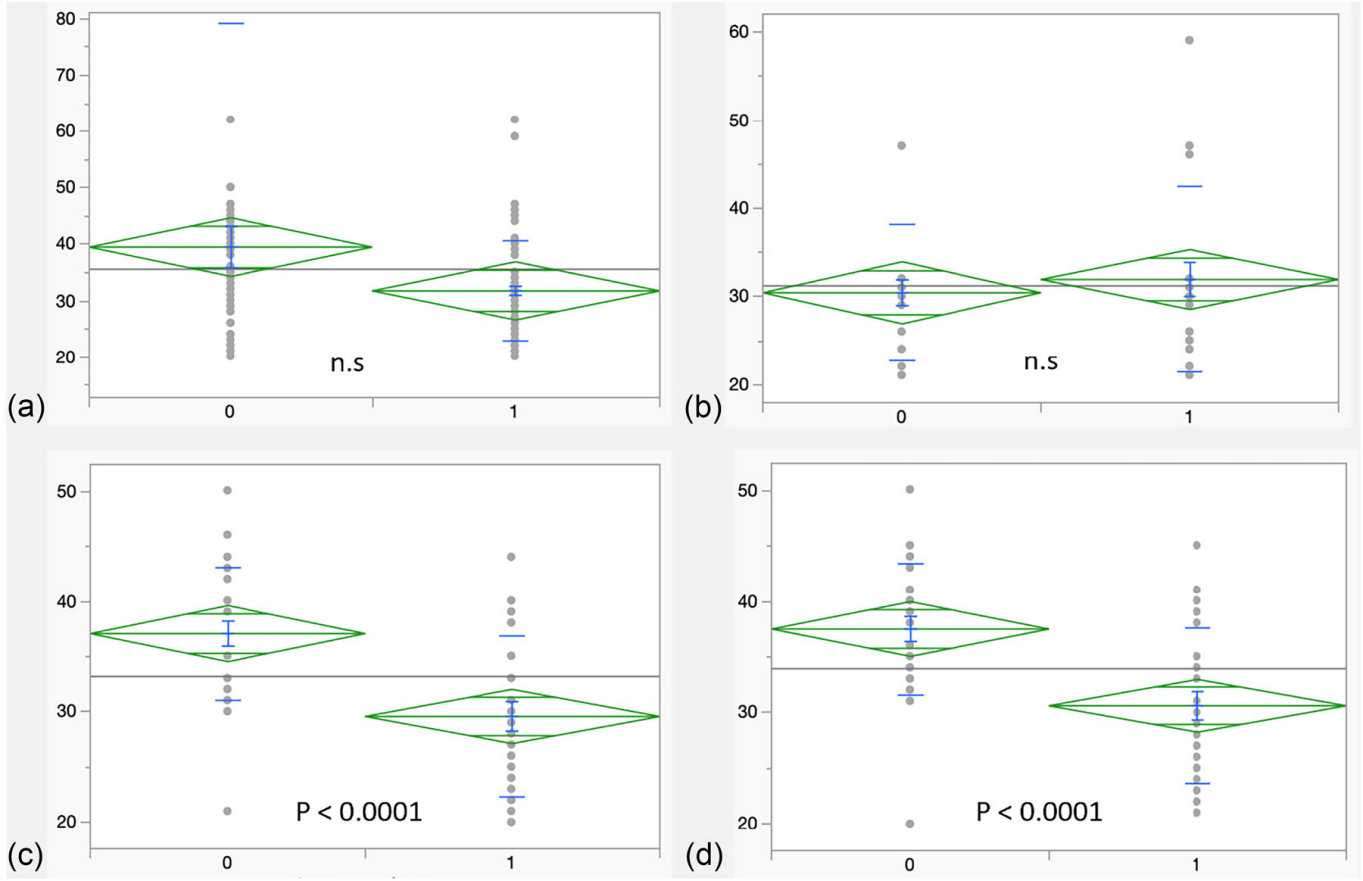
Plots demonstrating the difference in the STAI‐Y1 scores between the control group (0) and the VR group (1): (a) Preoperatively in the department. (b) Preoperatively in the anaesthetic room. (c) Intraoperatively during skin closure. (d) postoperatively in the anaesthetic room. STAI Y‐1, State‐Trait Anxiety Inventory; VR, virtual reality.

The preoperative STAI‐Y1 score was 33.3 ± 7.9 in the control group versus 34.9 ± 9.7 in the VR group (*p* = 0.8). Intraoperatively, there was significantly less anxiety STAI‐ Y1 score in the VR group of 29.6 ± 7.3 compared to the control group at a score of 36.9 ± 5.8 (*p* < 0.0001). This continued to be the case in the immediate postoperative period, with significantly lower anxiety score STAI‐Y1 in the VR group versus the control group, 30.6 ± 6.9 versus 37.3 ± 5.7, respectively (*p* < 0.0001) Table 3. The STAI‐Y1 scores difference is demonstrated in diagrams in the perioperative period Figure 2.

There was no significant difference in postoperative nausea (20% in each group, *p* = 1). VAS satisfaction assessed by both the anaesthetist and anaesthetic nurse was significantly higher in the VR group versus the control group. On anaesthesiologist evaluation, 80% of the patients in the VR group scored on the higher satisfaction scores (9 & 10) versus 36.7% in the control group (*p* < 0.0001), and on the anaesthetic nurse evaluation: 93.3% of the patients in the VR group scored on the higher satisfaction scores (9 &10) versus 46.7% on the control group (*p* < 0.0001). However, there was no significant difference in VAS satisfaction assessed by the orthopaedic surgeon and orthopaedic nurse (*p* = 0.3 and 0.4, respectively) Table 4.

**Table 4:** VAS satisfaction evaluation by Orthopaedic (Ortho.) Surgeon, Ortho. Nurse, Anaesthetist (Anaes), and Anaes. Nurse.

Satisfaction	Total	Control group	VR group	*p* values
VAS satisfaction (Ortho. Surgeon)				0.3
7	1	1	0	
8	2	1	1	
9	52	24	28	
10	5	4	1	
VAS satisfaction (Ortho. Nurse)				0.4
7	5	3	2	
8	5	2	3	
9	17	11	6	
10	33	14	19	
VAS satisfaction (Anaesthetist)				<0.0001
7	14	14	0	
8	11	5	6	
9	25	7	18	
10	10	4	6	
VAS satisfaction (Anaes. Nurse)				<0.0001
7	9	9	0	
8	9	7	2	
9	15	8	7	
10	27	6	21	

Abbreviations: VAS, visual analogue scale; VR, virtual reality.

At one year postoperatively, there was no significant clinical difference between both groups as reflected by the SKV scores of 84.3 ± 19.4 [95% CI: 80.3–91] in the VR group versus 85.6 ± 13.5 [95% CI: 76.6–92] in the control group (*p* > 0.05).

## DISCUSSION

The most important finding of the presented study is demonstrating the significant positive impact of virtual reality utilisation, during ACL reconstruction surgery, on both intraoperative adverse events, intraoperative and immediate postoperative patient's anxiety and the overall patient satisfaction.

In the presented study, there was a significant reduction in intraoperative patient anxiety when a VR headset was used compared to the control group. This was demonstrated by low intraoperative STAI‐Y1 scores of 29.6 ± 7.3 in the VR group versus 36.9 ± 5.8 in the control group. Additionally, an immediate postoperative STAI‐Y1 scores were significantly lower in the VR group versus the control group, 30.6 ± 6.9 versus 37.3 ± 5.7, respectively (both *p* < 0.0001). Moreover, anaesthetic evaluation of patient satisfaction demonstrated a significantly higher percentage of patients reporting high satisfaction in the VR group compared to the control group 93.3% versus 46.7%, respectively (*p* < 0.0001).

Despite the presented in‐between group difference in the STAI‐Y1 score, with significantly lower scores in the VR group, there was no significant difference in the VAS anxiety score between both groups. Nevertheless, VAS anxiety scale is a one dimensional measure that assess patient's anxiety in a more broad nonspecific way, capturing patient's all feelings at a one given moment [15, 46]. Therefore, VAS scores may be influenced by temporary emotions, distractions, or external factors. Whereas, the STAI‐Y1 score is a more structured and comprehensive evaluation, addressing different dimensions of anxiety with multiple questions [40, 41]. Hence, STAI‐Y1 is less prone to momentary fluctuations and provides a more stable measure of state anxiety.

Despite that STAI‐Y1 is a self‐reported questionnaire, assessors can still interpret or record responses differently based on how they perceive the patient's state, which is certainly reflected on the overall patient satisfaction score. Orthopaedic surgeons are more focused on structural outcomes; the mechanical and rehabilitative aspects of the procedure. Therefore, orthopaedic surgeons my underestimate the emotional anxiety aspect. Whereas, anaesthetists pay more attention to the anxiety aspects even for subtle cues such as body language and nervousness, hence they are more inclined to believe specific anxiety levels are valid. In the presented study, there was no difference in patient satisfaction between the control and VR groups when assessed by both orthopaedic surgeon and nurse (n.s). On contrary, there was a significantly higher satisfaction in the VR group compared to the control group when assessed by both the anaesthetist and anaesthetic nurse.

Peuchot et al. [35] in a preliminary study, evaluated the use of VR during total knee arthroplasty surgery and demonstrated no significant positive differences attributed to the use of VR. Similarly, Huang et al. [24] investigated the effect of immersive VR on intravenous patient‐controlled sedation during both hip and knee arthroplasty and demonstrated no significant differences with or without using the VR. However, in their study, the modified Quality of Recovery Survey (QoR‐40) [33], did not consider patient anxiety [24]. To our knowledge, there are no studies that investigated the impact of VR on intraoperative adverse events and patient anxiety during ACL reconstruction surgery.

On the contrary, in upper limb surgery, the use of VR demonstrated a promising positive impact on patient anxiety and overall surgical experience [23]. Another positive impact was reported for VR in creating an effective distractive environment for paediatric patients during cast removal with reflection on reduced anxiety [25].

There is an intricate interplay between anxiety and arthrogenic muscle inhibition (AMI), through altered pain perception, psychological stress response, and attentional bias, which concludes that increased anxiety exacerbates AMI [21, 38]. Heightened AMI can significantly impede rehabilitation following ACL reconstruction, and there are multiple interventions have been introduced to remedy quadriceps activation failure following ACL injury or reconstruction [39]. However, targeting the aetiology makes a more powerful impact. In the presented study, the use of a VR headset has resulted in significantly less anxiety throughout the perioperative process, which should improve the postoperative rehabilitation phase.

Addressing anxiety‐related factors alongside traditional rehabilitation regimes should optimise treatment outcomes and return to sports following ACL reconstruction [4, 6, 12]. Beischer et al. [6] demonstrated that, regardless of age, athletes who returned to the sport and athletes who had symmetrical muscle function were those with stronger psychological profiles.

Tharion and Kale [43] demonstrated that intraoperatively immersive experience was an effective and acceptable alternative to pharmacological sedation in patients undergoing knee arthroscopic surgery under spinal anaesthesia. This has been reflected in a higher satisfaction level and no difference in preoperative to postoperative anxiolytic effect. This further emphasises the results from the presented study with a series focused only on ACL reconstruction. Hence, the use of VR during ACL reconstruction would yield overall better outcomes.

The limitations of the presented study include the inability to demonstrate whether the statistically significant difference in the STAI‐Y1 score equates to the minimal clinically important difference (MCID) of the score. This is mainly due to the lack of literature reporting on the MCID for STAI‐Y1 score. A benchmark study has reported on MCID and its clinical relevance in knee surgeries [28]. Therefore, future studies should focus on identifying the MCID for STAI‐Y1 score, preferably in knee surgeries. Another limitation is that the anaesthesiologists providing the medications were not blinded, which might be a source of bias. However, the scores were collected blindly.

## CONCLUSION

Virtual reality‐augmented anterior cruciate ligament reconstruction results in significantly fewer intraoperative adverse events, significantly less intraoperative and less immediate postoperative patient's anxiety, and higher overall patient satisfaction when assessed by the anaesthetic team.

## AUTHOR CONTRIBUTIONS

All have participated in the content and design of the study have seen and agreed with the contents of the manuscript.

## CONFLICT OF INTEREST STATEMENT

All authors have no conflicts of interest to disclose.

## ETHICS STATEMENT

This trial was registered at the hosting institution (APHM registration number DGR‐2020‐1261). All patient on the trial had given an informed consent for contribution and research.

## Data Availability

Data used in the study are available upon appropriate request.
